# Acceptability, Adherence, and Provision Through Antenatal Care: Evidence on Multiple Micronutrient Supplementation in Pakistan and Nigeria

**DOI:** 10.3390/nu18071101

**Published:** 2026-03-30

**Authors:** Jennifer Busch-Hallen, Jennifer Ayoub, Kimberly B. Harding, Shabina Raza, Osita Okonkwo, Babajide Adebisi, Loloah Chamoun, Khawaja Masuood Ahmed, Fazal Majeed, Abdul Latif, Ladidi Bako-Aiyegbusi, John Uruakpa, Samuel Obasi, Rilwanu Mohammed, Asim Shahzad Qureshi, Huma Habib, Huma Chishti, Nkechinyere Adinoyi, Jane Ezeonu, Sarah Anugwa, Lara Nasreddine, Colin Beckworth, Nadine Crossland, Chowdhury Jalal, Alison Greig, Mandana Arabi, Sarah N. Rowe

**Affiliations:** 1 Nutrition International, Ottawa, ON K2P 2K3, Canada; jbuschhallen@nutritionintl.org (J.B.-H.); jayoub@nutritionintl.org (J.A.); lchamoun@nutritionintl.org (L.C.); cjalal@nutritionintl.org (C.J.); agreig@nutritionintl.org (A.G.); marabi@nutritionintl.org (M.A.); 2 Dalla Lana School of Public Health, University of Toronto, Toronto, ON M5T 3M7, Canada; kimberly.harding@mail.utoronto.ca; 3 Nutrition International, Islamabad 44000, Pakistan; sraza@nutritionintl.org (S.R.); ashahzad@nutritionintl.org (A.S.Q.); hchishti@nutritionintl.org (H.C.); 4 Nutrition International, Abuja 900108, Nigeria; ookonkwo@nutritionintl.org (O.O.); badebisi@nutritionintl.org (B.A.); nadinoyi@nutritionintl.org (N.A.); jezeonu@nutritionintl.org (J.E.); sanugwa@nutritionintl.org (S.A.); 5 Nutrition Wing, Ministry of National Health Services, Regulations and Coordination, Government of Pakistan, Islamabad 45500, Pakistan; nfapakistan@gmail.com; 6 Directorate General Health Services and Integrated Health Program of Khyber Pakhutunkhwa, Peshawar 25000, Pakistan; 7 District Health Office of Swabi, Swabi 23430, Pakistan; 8 Federal Ministry of Health and Social Welfare, Abuja 900242, Nigeria; 9 National Primary Health Care Development Agency, Abuja 900103, Nigeria; 10 Bauchi State Primary Health Care Development Board, Bauchi 740001, Nigeria; 11 Independent Researcher, Islamabad 25000, Pakistan; hhabib@nutritionintl.org; 12 Department of Nutrition and Food Sciences, Faculty of Agricultural and Food Sciences, American University of Beirut, Riad El Solh, Beirut 11-0236, Lebanon; ln10@aub.edu.lb; 13 Red Kite Consulting, Saskatoon, SK S7L 0J8, Canada; colin@redkiteconsulting.ca (C.B.); nadine@redkiteconsulting.ca (N.C.)

**Keywords:** antenatal care, multiple micronutrient supplementation, maternal nutrition, maternal supplementation, acceptability, adherence, provision, facilitators, barriers, pregnant women, Pakistan, Nigeria

## Abstract

**Background/Objectives:** Globally, momentum is building around antenatal multiple micronutrient supplementation (MMS), with evidence that it is as effective as iron–folic acid supplementation in preventing maternal anemia and more effective in improving birth outcomes. In line with the World Health Organization 2020 recommendation and as part of a broader implementation research project, this study examines MMS acceptability, pregnant women (PW)’s adherence practices and experiences, and facilitators and barriers to acceptability, adherence, and provision of MMS within public ANC services in Pakistan and Nigeria. **Methods:** Following introduction of MMS by the Government of Pakistan in April 2022 (Swabi District) and the Government of Nigeria in December 2023 (Bauchi State), mixed-methods research was conducted using cross-sectional surveys (one in each country), focus group discussions (6 in Pakistan, 9 in Nigeria), and in-depth interviews (7 in Pakistan, 10 in Nigeria) with PW, family members, and facility- and community-based healthcare providers (HCPs). **Results:** Findings in both settings showed that MMS is widely accepted, and almost all women (>97%) started consuming the MMS they received. Adherence levels, assessed using both pill-count and self-reported measures, exceeded 70%. In both countries, perceived benefits were identified as a key enabler to MMS acceptability and adherence among PW, whereas perceived negative effects acted as a barrier. Facilitators of MMS provision included trusting relationships between PW and HCPs, while delayed antenatal care (ANC) initiation, anemia screening, and limited agency of PW were identified as barriers. **Conclusions:** This study provides findings to inform MMS scale-up across public ANC platforms in two low- and middle-income countries and contributes to global evidence on context-specific considerations for MMS implementation.

## 1. Introduction

Enhancing maternal and newborn health in low- and middle-income countries (LMICs) requires a strong focus on adequate nutrition during pregnancy [1]. The increased nutrient requirements of pregnancy, combined with potentially pre-existing deficiencies among undernourished pregnant women (PW), can adversely affect the health of both mothers and their infants [2]. For instance, maternal micronutrient deficiencies have been shown to increase the risk of low birth weight (LBW) (<2500 g), preterm delivery (<37 weeks), small-for-gestational-age (SGA, <10th percentile weight-for-age) infants, and both maternal and perinatal mortality [3].

The World Health Organization (WHO) has recommended, since 1968, routine daily iron and folic acid (IFA) supplementation during pregnancy to prevent maternal anemia and improve birth outcomes [4,5]. However, there is growing evidence from clinical trials and meta-analyses over the past two decades that multiple micronutrient supplementation (MMS)—including the United Nations International Multiple Micronutrient Antenatal Preparation (UNIMMAP), which provides 15 essential vitamins and minerals in one tablet—confers benefits beyond those of IFA alone, particularly in LMICs [6]. The evidence indicates that antenatal MMS is more effective in improving birth outcomes (including LBW, SGA births, and preterm delivery), provides similar prevention against maternal anemia, is more cost-effective compared to IFA, and is safe for both mothers and infants [2,7,8,9,10,11,12,13].

Drawing on the growing body of evidence, the 2020 WHO update of the 2016 antenatal care (ANC) recommendations stated that “antenatal multiple micronutrient supplements that include iron and folic acid are recommended in the context of rigorous research (context-specific recommendation—research)” [14]. This context-specific recommendation included that implementation research (IR) be undertaken in LMICs considering a transition from IFA to MMS [14] in order to address evidence gaps and implementation considerations including context-specificity and implications related to anemia burden [14,15]. IR helps to understand how to effectively deliver evidence-informed interventions—such as antenatal MMS—in real-world settings [16] thereby guiding the transition to and scale-up of MMS within the ANC platform. It further enables the identification of barriers and challenges that hinder effective implementation and allows for the development and testing of strategies to address the identified hurdles [17]. This is especially important given the long-standing problems implementing IFA supplementation, which have limited its potential for impact [18].

Globally, South Asia and Sub-Saharan Africa remain the regions with the highest burden of maternal undernutrition, including micronutrient deficiencies and anemia, and substantial challenges in maternal and newborn health [1,19,20]. In Pakistan, a South-Asian country, the prevalence of LBW (23% in 2018) and the burden of neonatal mortality (42 per 1000 live births in 2017–2018) [21] rank among the highest globally. According to the 2018 National Nutrition Survey in Pakistan, 42.7% of women of reproductive age (WRA) are anemic, 18.2% have iron deficiency anemia, 79.7% have vitamin D deficiency, 22.4% have vitamin A deficiency, and 14.4% are underweight [22].

Similarly, in Nigeria, a country in Sub-Saharan Africa, the prevalence of LBW is estimated at 7%, based on pooled analyses of the 2013 and 2018 Nigeria Demographic and Health Surveys (NDHS) [23], and neonatal mortality rate remains high (39 deaths per 1000 live births in 2018) [24]. In addition, nationally representative data from the NDHS 2018 showed that 58% of WRA are anemic and 12% are underweight [24], and the 2021 Nigeria National Food Consumption and Micronutrient Survey (NFCS-MN) reported that 11.6% have vitamin A deficiency [25].

As part of efforts to address maternal anemia, both Pakistan and Nigeria have implemented IFA supplementation within routine ANC [25], although multiple implementation bottlenecks and barriers have been reported [18,26,27]. Gaps in IFA adherence are also a documented issue; for instance, the proportion of PW who took IFA for at least 90 days of their pregnancy was estimated at 29% in Pakistan [20] and 31% in Nigeria [24]. This is concerning, since regular adherence to micronutrient supplementation throughout pregnancy is necessary to achieve the benefits demonstrated through research [28]. A 2025 meta-analysis found that among women taking MMS, those achieving ≥90% adherence showed significantly greater improvements in birthweight and reductions in LBW (compared to IFA) than those with lower adherence [28].

In its 2022–2027 Maternal Nutrition Strategy, the Government of Pakistan (GoP) stated its commitment to addressing the critical maternal nutrition situation in the country and included a recommendation to integrate MMS within its ANC services [29]. Similarly, in 2021, the Government of Nigeria (GoN) approved the use of MMS during pregnancy [30], and the National Guidelines for the Prevention and Control of Micronutrient Deficiencies in Nigeria indicated that MMS is intended to replace daily IFA supplementation for all PW [31]. Accordingly, MMS was introduced through public ANC services in selected areas of Pakistan in April 2022 and in selected regions of Nigeria in December 2023. Both countries use the UNIMMAP formulation, which contains 15 vitamins and minerals, including 30 mg of iron [6]. Nutrition International collaborated with the GoP (Nutrition Wing of the Ministry of National Health Services, Regulations and Coordination, Islamabad, and the Directorate General Health Services and Integrated Health Project of the Health Department Government of Khyber Pakhtunkhwa and the District Health Office of Swabi) and the GoN (Federal Ministry of Health and Social Welfare, National Primary Health Care Development Agency, Bauchi State Primary Health Care Development Board) on IR in the Swabi district (Pakistan) and Bauchi State (Nigeria) to identify effective implementation approaches and inform the sustainable transition to MMS as part of public ANC in these two settings. The selection of Swabi district in Pakistan was guided by various considerations, including an established research infrastructure, strong existing maternal nutrition programming, accessibility, safety, and government readiness. Similarly, the selection of Bauchi State in Nigeria was informed by government readiness and implementation infrastructure, including a foundation of strong maternal nutrition programming, as well as the importance of focusing on northern Nigeria from an equity perspective [32]. Bauchi’s religious diversity within a single state also provided an opportunity to capture varied sociocultural contexts [33].

As part of these broader IR projects, the present mixed-methods study explored the introduction of MMS in Pakistan and Nigeria and aimed to examine MMS acceptability, barriers and enablers to MMS provision, as well as PW’s MMS adherence practices and experiences. In addition to PW and HCPs, family members were included in the study, recognizing that women’s autonomy is often limited in many low- and middle-income settings, including Pakistan and Nigeria, and that decisions about ANC and supplement use are frequently made at the household level [34,35]. In both Pakistan and Nigeria, husbands play a central role in maternal health decision-making; therefore, their engagement was prioritized in both countries [34,35]. In Pakistan, as in other South Asian settings where mothers-in-law are widely recognized as key influencers of pregnancy-related decisions and maternal health behaviors, mothers-in-law were also invited to participate in the study [34,35].

## 2. Materials and Methods

### 2.1. Study Setting and Design

In Pakistan, beginning in April 2022 and through collaboration among federal, provincial and district health officials, MMS was integrated into routine preventive ANC services within the public sector across the Swabi district of Khyber Pakhtunkhwa province. With a population of 1.89 million in 2023, Swabi is among the largest districts of Khyber Pakhtunkhwa; across this province, approximately 85% of the population reside in rural or remote areas [36], and 33% of women of reproductive age are anemic [22]. Once MMS was introduced in Swabi district, all PW accessing public ANC services were offered up to two bottles of MMS (UNIMMAP formulation, 100-count) over the course of their ANC, whether at health facilities or within the community. This new standard of care replaced the provision of IFA (~10–30-count, mixed packaging) across multiple ANC visits for the prevention of anemia.

In Nigeria, beginning in December 2023, MMS was integrated as the new standard of care within routine preventive ANC services in three local government areas (LGAs)—Dass, Ganjuwa, and Giade—of Bauchi State, where the prevalence of anemia among WRA exceeds the national estimate (68.6% vs. 58.0%) [24]. Since then, all PW accessing ANC services in public health facilities in these three LGAs were provided up to two bottles of MMS (UNIMMAP formulation, 100-count) instead of IFA supplements (typically uncombined, i.e., separate iron and folic acid tablets, 30-count across multiple ANC visits).

In both Pakistan and Nigeria, MMS was provided through the same delivery platforms previously used for IFA, including routine facility-based ANC and community-based distribution where applicable. To further support the introduction of MMS in both countries, training activities for the health workforce were conducted along with implementing MMS monitoring tools, and strengthening the ANC platform through supply chain management, including forecasting, microplanning, stock management, and distribution. In each country, a Technical Working Group was convened to oversee the broader IR, including the present study.

Within this context, and as part of a broader IR project, the present study adopted a triangulation (convergent) mixed-methods design, in which the quantitative and qualitative components were conducted during the same implementation period, analyzed separately, and integrated at the interpretation stage, with neither component dependent on the results of the other [37]. The quantitative component involved cross-sectional surveys and assessed MMS acceptability among key stakeholders, as well as adherence and adherence practices among PW. The basic qualitative research [38] involved focus group discussions (FGDs), and interviews, with the aim of exploring facilitators and barriers related to MMS acceptability, adherence, and MMS provision—focusing on individual-level, family/community-level, and system-level factors. The qualitative component, which used both FGDs and IDIs, was intended to capture complementary dimensions of MMS experience and adherence and to enable triangulation across both sources. FGDs explored shared norms, collective perceptions, and community dynamics influencing supplement use, while IDIs allowed for deeper exploration of individual experiences, decision-making processes, and potentially sensitive issues [39]. Sampling for the qualitative component was conducted independent of that for the quantitative survey component.

### 2.2. Study Population

#### 2.2.1. Pakistan

Survey

From the 53 administrative Union Councils (UCs) of Swabi, 24 UCs were eligible based on the following criteria: absence of major security concerns, availability of female HCPs (as women in Swabi typically do not seek ANC from male providers due to cultural norms), and the presence of at least five potentially eligible HCPs. All 24 UCs were included in the study. Four types of participants were included in the survey: HCP, PW, husbands of PW, and mothers-in-law (MIL) of PW. For the HCPs, two types of provider units, who provide both ANC and MMS to PW, were sampled: (1) primary public health facilities (staffed by Women Medical Officers (WMO) and Lady Health Visitors (LHV)), and (2) community-based providers (Lady Health Workers (LHWs)). In each UC, 70% of the HCP units were randomly selected from the available list of HCPs units, resulting in the selection of 280 HCPs units in total.

As HCPs maintain a list of PW who have been provided with MMS each month, the sampling of PW for the survey was conducted using HCP records. Eligible women were pregnant or up to three months postpartum, and had received their first bottle of MMS at least 30 days earlier from a participating public healthcare facility or community-based LHW. From the health records of each selected HCP unit (for the period of December 2022–February 2023), five pregnant or postpartum women who had received at least one bottle of MMS were randomly chosen, yielding a target sample of 1400 women.

Eligible family members of PW included husbands and MIL or another influential female family member (IFFM) in case the MIL was not available.

Qualitative component

Data collection was conducted in 3 UCs, which were selected to provide diversity in provision of MMS at the healthcare facility and community levels. Data was collected from HCPs and PW. HCPs were purposively sampled and recruited from the health facilities within the selected UCs as well as from the LHWs designated in those catchment areas, for in-depth interviews (IDIs). Each participating HCP was asked to provide information for 1–2 potentially eligible women to participate in FGDs. Eligible women were those who had received their first MMS bottle at least 30 days earlier and were either in the advanced stages of their pregnancy or within three months postpartum. PW were recruited among clients of community-based HCPs in settings with low and high MMS bottle uptake, as well as among clients of facility-based care providers in facilities with low and high bottle uptake. Women were purposively sampled so they could draw on a longer timeframe of MMS experience.

#### 2.2.2. Nigeria

Survey

The study was conducted in three LGAs of Bauchi State (Dass, Ganjuwa, and Giade), within which 32 wards were randomly selected. Within each selected ward, all eligible primary health facilities and secondary health facilities with an average of at least 15 monthly first-ANC attendance were selected. A list was compiled of all eligible PW or women in the postpartum period (up to 3 months postpartum) who had reportedly received at least one MMS bottle for at least 30 days between the commencement of MMS distribution and the start of data collection in participating facilities. From this list, a random sample proportional to the number of PW in each ward was drawn yielding a target sample size of 928 women. Husbands of the enrolled women were also included in the study.

Qualitative component

Data for the qualitative component was collected in the three same LGAs in Bauchi State using purposive sampling. Study participants included PW or women in the postpartum period (up to 3 months postpartum) attending the selected health facilities, who had received MMS. In addition to eligible women, participants also included husbands and HCPs. Women who had attended at least two ANC sessions at the health facility and had received at least one bottle of MMS were purposively selected for the interviews and FGDs. To capture varying perspectives, these women were sampled to include diversity in key factors that may be related to MMS experience including age, literacy levels, ANC utilization and pregnancy status. Similarly, husbands of women participating in the study were selected, and HCPs who provide both ANC services and MMS to PW were purposively selected for IDIs and FGDs.

### 2.3. Data Collection

#### 2.3.1. Pakistan

In Pakistan, the cross-sectional survey was conducted from 17 March to 4 April 2023. Data was collected using interviewer-based questionnaires administered by local enumerators who had relevant prior survey experience and underwent training before the start of data collection. There was one questionnaire for each participant group (HCPs, PW, husbands, and MIL/IFFM). A pretested, multicomponent questionnaire, which was designed by the research team, was used. The questionnaires were developed in English, translated into Urdu, and the translation was validated by bilingual study staff through comparative review of both versions during the pre-testing phase. The questionnaires inquired about socio-economic characteristics, acceptability of MMS (based on perceived importance and perceived benefits) among PW, husbands, and MIL/IFFM; acceptability of switching to MMS among HCPs; adherence to MMS among PW based on self-reported data, as well as adherence practices (including whether they stopped taking MMS and reasons for doing so). The enumerators also manually counted any remaining MMS tablets using a pill-counter designed for the purposes of this study.

The data collection tools were designed in a computer-assisted personal interviewing (CAPI) format and administered on Android devices using the KoBo Toolbox application Version 2.023.37. For quality control, the digital tool was linked to a structured database and equipped with automated error detection and consistency check mechanisms for key variables, thus minimizing data errors and enabling timely resolution of discrepancies. Additional quality control measures included field visits and field team consultations as well as a review of collected data at regular intervals.

For the qualitative component, IDIs and FGDs were conducted in November 2023. IDIs were held with 7 HCPs of different cadres (facility-based LHV, WMO, and community-based LHW). The number of FGDs and IDIs was determined a priori based on project timelines and logistical constraints rather than being driven by thematic saturation. An IDI guide was used to guide the interview discussions. Six FGDs were conducted with PW: 3 among clients of community-based HCPs (1 in setting with low bottle uptake, 2 with high uptake), and 3 FGDs with clients of facility-based care providers (1 in a facility with high bottle uptake and 2 with low bottle uptake). Each FGD was conducted with 6 PW. A FGD guide was used to guide the discussions. All interviews and discussions were conducted in the local language. Following consent, the IDIs and FGDs were audio-recorded, supported by detailed notes taken in real-time.

#### 2.3.2. Nigeria

In Nigeria, the cross-sectional survey was conducted in March 2024. The survey tools were developed by adapting the questionnaires used in Pakistan and targeted PW and husbands. Following this adaptation, the questionnaires were translated into Hausa. The Hausa translation was validated through a comparative review of the English and Hausa versions conducted by bilingual study staff during pre-testing. Both the English and Hausa versions were programmed onto the SurveyCTO version 2.60 platform for use on Android-based tablets. This programming included quality control features such as capturing GPS coordinates of interview locations and enabling audio recordings of interviews, along with meta-data like the time spent per question. After programming, the English and Hausa versions of the tools were pretested, and feedback from the pretesting was used to update both versions before the full survey rollout. Data was collected through face-to-face interviews administered by trained field workers using SurveyCTO. The platform uses transport layer security (TLS) to sync data to a secure SurveyCTO server. The collected meta-data enabled high-frequency checks to flag any suspect interviews, and the audio recordings were reviewed for quality assurance. These measures—combined with field monitoring, supervision, daily performance tracking via a matrix and WhatsApp, regular data checks, back-check surveys, and geo-spatial analysis—ensured that enumerators adhered to their assigned wards and followed the survey protocol.

The qualitative component included FGDs and IDIs conducted in March–April 2025, moderated using study-specific interview guides and conducted in local language. In total, three IDIs were conducted with PW, three with HCPs, and four with husbands. Four FGDs were held with PW, one with HCPs, and four with husbands. Each FGD comprised 6–8 participants. Following consent, the IDIs and FGDs were recorded with hand-held devices (audio-recorders). Data collection continued until thematic saturation was reached.

### 2.4. Ethical Approval

In Pakistan, the study was approved by the Ethical Review Committee (ERC) of the Health Services Academy Islamabad and oral informed consent was obtained from all study participants. In Nigeria, ethical approval was obtained from the National Health Research Ethics Committee as well as the Bauchi State Health Ethics Committee. Due to literacy limitations, oral consent was obtained from all participants.

### 2.5. Data Analysis

For the quantitative data, descriptive statistics were computed, including means, frequency, distributions, and proportions with 95% confidence intervals. These were analyzed using Stata (version 17.0) and IBM SPSS Statistics (version 31). The assessment of adherence was based on the self-reported number of MMS tablets consumed during pregnancy (for women who were pregnant or postpartum at the time of survey), as well as on pill count (for women who were pregnant at the time of survey only). For the latter, the number of MMS bottles given to PW was recorded; remaining pills were counted as part of the data collection. Percent adherence to the daily regime was calculated as the proportion of days MMS was consumed out of the total number of days the woman was expected to consume MMS (i.e., based on the number of days between the day the woman received the first bottle of MMS and the day of the survey for pregnant women or the end of pregnancy for postpartum women). In addition, the proportions of PW who consumed at least 75% and 90% of the expected MMS tablets were also calculated.

For the qualitative component, data from FGDs and interviews were translated to English and analyzed using an inductive thematic approach [40]. Each transcript was first manually coded line by line, after which codes were organized into overarching themes and sub-themes [41]. An Excel matrix was used to compile and group codes by theme. The analysis followed several steps: (1) Familiarization: conducted via multiple reviews of transcripts and codebooks; (2) Code generation: with each review of the data, thematic codes were generated and refined; (3) Themes identification: classification of data into themes arising inductively from the transcripts; (4) Review and modification of codes and themes; (5) Systematic examination of coded data within and across themes to identify patterns, relationships, and linkages that inform key considerations and recommendations. Coding, thematic categorization, and data interpretation were primarily conducted by one data analyst, with a second analyst independently reviewing a subset of transcripts to verify the coding. Any discrepancies were resolved through discussions. Both analysts had professional backgrounds in public health and implementation research and reviewed and validated the final themes in consultation with a broader working group. The analysts were not involved in direct service delivery to participants.

## 3. Results

### 3.1. Characteristics of Survey Population

In Pakistan, the final sample for the survey consisted of 259 HCPs, 1166 women, 703 husbands, and 929 MILs. In Nigeria, the survey sample included 950 PW and 254 husbands.

Table 1 presents the socio-demographic and ANC characteristics of women participating in the surveys. In both countries, the sample of PW was predominantly in the range of 20–29 years, and the proportions of those with no formal education ranged between 44.0% in Pakistan and 65.6% in Nigeria. The majority of women were pregnant at the time of the survey (73.2% in Pakistan and 68.7% in Nigeria). Women’s healthcare decisions in Pakistan were largely made by themselves (42.8%) and their husbands and MILs (54.9% and 49.8%, respectively), while in Nigeria, husbands were the main decision-makers (92.4%) (Table 1). The majority of PW in Pakistan lived with their extended families (84.3%), while in Nigeria, this proportion was close to 40%. On average, PW had three to four ANC visits in their most recent pregnancy. In Pakistan, 91.9% of women received MMS from a LHW (community-based HCP); around two-thirds of the time (69.7%), MMS was provided at the first ANC contact. While in Nigeria, most women (81.6%) received their MMS from a CHEW or JCHEW (facility-based HCP), usually at their first ANC visit (92.0%).

### 3.2. Acceptability of MMS Among PW and Key Stakeholders

#### 3.2.1. Survey Findings

As shown in Table 2, the proportions of PW who described taking MMS every day as “important” or “very important”, ranged between 93.1% in Pakistan and 98.5% in Nigeria. Similarly, the large majority of husbands in both countries (97.5% in Pakistan and 93.7% in Nigeria), as well as MILs in Pakistan (82.3%) agreed that it is “important” or “very important” for PW to take MMS every day. The proportion of PW who were able to describe at least one benefit of MMS was estimated at 91.3% in Pakistan and 81.5% in Nigeria, while the proportion of husbands who could describe at least one benefit was 100% and 69.7%, respectively. In Pakistan, 75.1% of MILs were able to describe at least one benefit of MMS, while 67.8% of HCPs were able to describe at least three MMS benefits. In addition, the proportion of HCPs who described the transition to MMS as “positive” or “very positive” was estimated at 91.5% in Pakistan.

#### 3.2.2. Facilitators and Barriers to MMS Acceptability

The qualitative findings identified facilitators and barriers to acceptability of MMS among key stakeholders in both Pakistan and Nigeria (Table 3). In both countries, facilitators included perceived benefits of MMS, with participants describing improvements in perceived health and relief from pregnancy-related symptoms as key drivers of acceptability. Another common facilitator in both settings was MMS financial accessibility. Additional enablers identified in Pakistan included ease of access to ANC services at or near the home, as well as trust in the source recommending MMS, whereby reassurance from trusted individuals (family members and HCPs) helped address initial doubts about the supplement. In Nigeria, physical and sensory properties of the MMS tablets were identified as a facilitator: HCPs reported that women appreciated having to take fewer tablets (i.e., one tablet of MMS in comparison to two tablets of iron and folic acid), as well as the improved pill properties, including less odor compared to IFA.

In both countries, barriers to MMS acceptability included perceived negative effects, as some women believed that MMS could cause unwanted side effects or adverse pregnancy outcomes, thereby limiting its acceptability. In both settings, prior positive pregnancy experiences without the use of MMS lowered the perceived need for supplementation. Additionally, in Pakistan, mistrust in the system providing MMS was identified as an additional barrier to acceptability. For instance, one HCP indicated that some women were hesitant about free, government-distributed products, which undermined confidence in MMS for some PW.

### 3.3. Adherence Among PW

#### 3.3.1. Survey Findings

All women enrolled in this study received at least one bottle of MMS, and almost all started consuming the tablets (97.4% in Pakistan and 99.9% in Nigeria) (Table 4). In both countries, PW consumed 73.6% of the expected tablets, based on pill count. Using this measure, 60.4% of women in Pakistan and 55.1% in Nigeria consumed at least 75% of the expected tablets; 48.6% and 40.1%, respectively, consumed at least 90%. When using self-reported data among all women (PW and post-partum), daily adherence to MMS was 76.9% in Pakistan and 72.5% in Nigeria. The proportion of women who reported consuming at least 75% of expected tablets was 66.3% in Pakistan and 55.4% in Nigeria, and those who reported consuming at least 90% of expected tablets was 57.3% and 41.4%, respectively.

Further analysis of self-reported data among the subgroup of PW only, yielded estimates of 71% and 73.2% for daily adherence in Pakistan and Nigeria, respectively. In addition, the proportions of PW who reported to consume at least 75% of expected tablets were 56.8% in Pakistan and 53.1% in Nigeria, while those who reported to consume at least 90% were 45.6% and 36.4%, respectively.

In both countries, approximately one third of women (28.3% in Pakistan and 35.2% in Nigeria) reported they stopped taking MMS at some point during their pregnancy; with the majority (about 73% in Pakistan and 76% in Nigeria) resuming to take MMS soon thereafter, within 6–7 days. The most common reasons for stopping MMS were forgetting (62.1% in Pakistan and 72.5% in Nigeria) and perceived side effects (36.6% in Pakistan and 11.1% in Nigeria).

#### 3.3.2. Facilitators and Barriers to Adherence Among PW

In both Pakistan and Nigeria, perceived benefits facilitated MMS adherence, with participants describing symptom relief and the desire to achieve positive pregnancy outcomes as key motivators for taking MMS (Table 5). The use of daily reminder techniques, including visual and time- and place-based cues, as well as encouragement by family members were also identified as facilitators of MMS adherence in both countries. In Pakistan, additional facilitators included knowledge related to MMS and practical guidance from HCPs on MMS including strategies to mitigate side effects. In Nigeria, fear or shame-based beliefs about lack of adherence appeared to facilitate adherence. For instance, some respondents believed that not taking MMS would be identifiable by HCPs at the time of delivery such as delivering an ‘unclean’ baby, hence motivating adherence among PW.

In both countries, perceived negative effects emerged as a key barrier to adherence. Some participants expressed fears that MMS use could result in negative outcomes, including nausea or having large babies, which contributed to hesitancy and reduced adherence. An additional barrier identified in Pakistan was related to disruptions to daily routines, with PW describing forgetting to take MMS when their usual routines were interrupted.

### 3.4. Provision of MMS

#### Facilitators and Barriers to the Provision of MMS

Qualitative findings helped identify several factors that drive the provision of MMS through ANC (Table 6). In both countries, open communication and a trusting relationship between HCP and PW were identified as facilitators of MMS provision. Participants described preferring to receive MMS from trusted HCP, particularly when providers offered clear explanations, ongoing support, and accessible follow-up. In addition, in both countries, the accessibility of MMS was identified as a facilitator with participants in Pakistan describing how the availability of ANC and MMS close to their home or in their community reduced time, mobility, and sociocultural barriers that might otherwise limit access to MMS, and in Nigeria availability and accessibility of MMS within the facility was highlighted as an enabler. In Nigeria, a facilitator specific to the provision of MMS was HCPs’ training and their recognition that training improved their delivery of MMS.

In both countries, heavy workload among HCPs was identified as a barrier to MMS provision (Table 6). Participants described how high patient volumes limited the time available for providing and counseling on MMS. Also in both countries, anemia diagnosis and management were perceived as a barrier to MMS provision. In Pakistan, delays in anemia screening (because of lack of laboratory capacity at the community level and within some facilities), restricted opportunities for early detection of anemia and, given the anemia management guidelines at the time of the study, this delayed timely access to supplementation. Similarly, in Nigeria, the need for anemia screening was mentioned by some HCPs as a factor that may restrict or delay provision of MMS given that anemia management guidelines at the time of the study did not include MMS for anemic PW. In addition, in both countries, limited agency among PW to make health-related decisions was identified as a barrier to MMS provision. In Pakistan, HCPs reported that household decision-making dynamics sometimes restricted women’s ability to attend follow-up visits, thereby disrupting access to subsequent MMS, and in Nigeria, limited family support, coupled with logistical constraints such as transportation appeared to be barriers to MMS provision. Delayed initiation of ANC was noted as a barrier in both countries, with participants describing late presentation of PW to ANC services, which results in PW receiving MMS late in pregnancy. Sociocultural norms were also identified as barriers to MMS provision in both countries. In some settings in Pakistan, prevailing norms discourage women from disclosing their pregnancy to family members, which can delay access to ANC services and the provision of MMS. In Nigeria, belief in traditional healing practices was noted as a barrier to healthcare-seeking from formal facilities, hence limiting opportunities for MMS provision. Though MMS supply generally appeared to not be an issue, some participants in Pakistan and Nigeria mentioned delays in the supply chain or stock outs. In Pakistan, participants also described limited communication and coordination among and between different cadres involved in ANC service delivery (such as facility-based vs. community-based), contributing to gaps in information flow and the continuity of care, which may also affect provision of MMS.

### 3.5. Overview of Facilitators and Barriers to MMS Acceptability, Adherence and Provision

**Figure 1:**
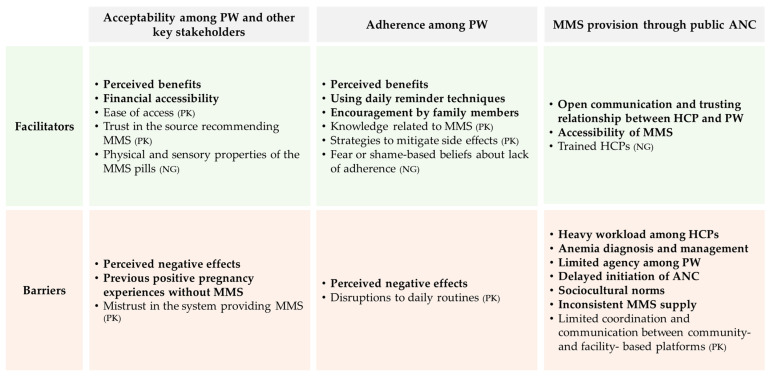
Summary of common and country-specific themes affecting MMS acceptability, adherence, and provision in Pakistan and Nigeria. **Bold** text denotes factors common to both Pakistan and Nigeria; country-specific factors are indicated by country abbreviation. Abbreviations: ANC: antenatal care; HCP: healthcare providers; MMS: multiple micronutrient supplementation; NG: Nigeria; PK: Pakistan; PW: pregnant women.

Figure 1 provides a summary of the identified facilitators and barriers to MMS acceptability, adherence, and provision in both Pakistan and Nigeria, while also presenting the commonalities between the two countries.

## 4. Discussion

This study provides evidence from initial implementation of antenatal MMS in Pakistan and Nigeria and included insights on acceptability among PW and other key stakeholders, barriers and enablers of MMS provision to PW, and PW’s adherence practices and experiences.

Daily adherence levels in Pakistan (76.9% (self-reported), 73.6% (pill count)) surpassed available estimates of IFA adherence at the sub-national and national levels, although variation in adherence definitions across studies may limit direct comparability. For instance, in a study conducted among PW in the neighboring district of Peshawar (Pakistan), Ummair et al., 2025 reported that 34.4% of women took at least four IFA tablets per week [42]; while the National Nutrition Survey (2018) [22] showed that only 22.2% of women consistently took IFA supplements for 90 days or more. Similarly, MMS adherence levels in Nigeria (72.5% (self-reported), 73.6% (pill count)) exceeded those reported for IFA by the Nigeria Demographic and Health Survey 2018 [24] where 31% of women reported taking IFA for at least 90 days, as well as estimates from a regional/multi-country analysis in Sub-Saharan Africa (pooled prevalence of compliance to IFA of 39.2%) [43]. The relatively high MMS adherence levels observed in our study may, in part, be attributable to the government-led rollout, which included improvements to the ANC system beyond what was in place for IFA (i.e., strengthened supply mechanisms and HCP training focused on enhancing skills and knowledge to effectively deliver MMS). In addition, the observed adherence levels, which surpassed those reported for IFA, are consistent with evidence from several studies in LMICs indicating that MMS is often preferred over IFA among PW, particularly with respect to its organoleptic properties, perceived benefits, and side-effect profile [44,45,46,47,48,49,50].

In line with previous studies [44,45], our findings showed that MMS is highly acceptable to PW in both Pakistan and Nigeria, with more than 90% stating the importance of daily MMS intake and over 80% being able to describe at least one perceived benefit. These responses reflect positive cognitive and emotional dimensions consistent with the definition of high acceptability [44], which in turn is recognized as a key determinant of sustained adherence [44]. Qualitative findings further identified perceived benefits among PW—such as relief from pregnancy-related symptoms, increased appetite, and enhanced physical strength—as key facilitators of both MMS acceptability and adherence in Pakistan and Nigeria. Many of these perceived benefits and their positive associations with acceptability and adherence have similarly been described by PW in other settings, including Mali, Niger, Jordan, and Indonesia [44,46,47,51]. The packaging and format of MMS appeared to also facilitate adherence, as MMS was provided as a single daily tablet in a 100-count bottle [49], in contrast to IFA, which was typically distributed in smaller, less consistent quantities. In Nigeria, the iron and folic acid were also provided as two separate tablets. Furthermore, financial (no-cost) accessibility emerged as a facilitator of MMS acceptability in our study. This is consistent with evidence from Ethiopia, where Abebe et al. (2025) identified the provision of MMS at no cost as an enabler of overall acceptability [45].

In addition to PW, our survey results and qualitative findings showed that MMS was highly acceptable to influential family members, with husbands expressing positive perceptions of MMS, such as its role in improving women’s strength, ability to perform daily activities, and overall health, as well as in reducing pregnancy-related complications. In settings such as Pakistan and Nigeria, where healthcare decisions pertinent to women are often made by their husbands and other influential family [52,53], our findings highlight the importance of leveraging family engagement to foster acceptability and support for PW [54].

Despite the high acceptability and overall adherence, the proportion of women who consumed at least 75% of the expected MMS tablets was in the range of 55–60%, and those who consumed at least 90% did not exceed 50% in either country. These findings highlight the need for continued efforts to further improve adherence, as MMS adherence above 75% is associated with improvements in birth outcomes compared to IFA, with even greater benefits for adherence ≥90% [28]. More specifically, high adherence to maternal MMS (defined as ≥90% adherence) compared to IFA was significantly associated with higher birthweight (+44 g; 95% CI: 31–56) and lower risks of LBW (RR 0.93; 95% CI: 0.88–0.98) and SGA (RR 0.95; 95% CI: 0.93–0.98), whereas lower adherence (<60%) showed none of the birth outcome advantages relative to IFA [28].

Our findings therefore underscore the need to build on existing enablers and address prevalent adherence barriers among PW to foster sustained MMS use. In our study, 29% of women in Pakistan and 35% in Nigeria reported that they stopped taking MMS at some point during their pregnancy, with the main reason for stopping being attributed to forgetting (62.1–72.5%). This finding is in line with the literature [44,45] and underscores the need to develop strategies to support women to remember to take MMS every day [55]. In this context, our qualitative data helped to identify potential strategies to help PW remember to take MMS daily. In both Pakistan and Nigeria, a common facilitator of adherence was the adoption of habit-forming strategies, including the use of aide-memoires and visual cues as reminders (i.e., keeping bottle in a visible location). Addressing one of the identified barriers to provision—late initiation of ANC—is also central to optimizing adherence. To maximize the benefits of MMS, women need to begin ANC early in pregnancy so they can receive and start taking MMS as soon as possible. Therefore, strengthening ANC attendance, especially early initiation, is an important area of focus for efforts to improve MMS adherence. Previous studies have shown that family support and engagement—particularly through adherence partners such as husbands—the use of tracking tools (e.g., calendars), and broader community-based approaches, including community health worker home visits, women’s groups, and community mobilization, represent acceptable, low-cost strategies with strong potential to improve both antenatal micronutrient supplement adherence and early ANC engagement in LMIC settings [54,56,57,58,59].

In addition, our results showed that, of those who stopped taking MMS, 11.1% in Nigeria and 36.6% in Pakistan attributed discontinuation to perceived side effects. Our qualitative data further showed that perceived lack of benefits or negative effects, including side effects, were a common barrier to both adherence and acceptability among PW in both countries. Several studies have however suggested that the negative side effects attributed to MMS may in fact be stemming from pregnancy itself rather than the use of the supplement [47,49]. In addition, when MMS-related side effects are reported by PW, evidence indicates that counseling on side-effect management and the provision of simple instructions—such as taking supplements with meals or before bedtime—can help facilitate and improve adherence, highlighting the need for interventions to strengthen MMS counseling as part of ANC [44]. For instance a previous quasi-experimental study conducted in Ethiopia showed that counseling sessions based on the health belief model, and including increasing PW’s knowledge of undernutrition and its consequences, how to take the supplement, when to take it, common side effects and their management; resulted in significantly improved IFA adherence [60].

This study has also identified key facilitators and barriers to MMS provision. It is important to note that we consider “provision” as going beyond simply handing PW an MMS bottle. It includes both the physical distribution of MMS within ANC services (with adequate supply, monitoring systems, and a trained workforce), as well as the delivery of women-centered care according to standards (whereby PW receive their first MMS bottle at their first ANC contact coupled with high-quality counseling at initial and subsequent visits). This is in line with the model for measuring effective coverage, which highlights that the quality of coverage (or provision) of an intervention is a critical step along the pathway towards achieving the intended health outcomes [61]. Key facilitators of MMS provision for both countries included open communication and trusting relationships between HCPs and PW. This finding aligns with evidence from previous studies highlighting trust in HCPs and supportive counseling as among the primary facilitating factors for improving supplement delivery and use [44]. For example, a study conducted in Niger showed that supplementation was positively perceived by PW because of the high level of trust in medical providers [46]. HCPs play a critical role in facilitating supplementation by providing information about the supplement, addressing concerns, and counselling PW and their families on the benefits and use [62]. In line with available evidence [44], another common enabler to MMS provision in both countries was the accessibility of MMS to PW. In Pakistan, PW reported that home delivery by community-based LHW enhanced access and hence acceptability of the supplements, and in Nigeria, accessibility of MMS within facilities was also positively perceived. This finding is particularly relevant for other settings where community-based HCPs play a role in ANC service delivery, especially in contexts where women have limited access to transportation and restricted mobility [53].

Barriers to MMS provision were also linked to anemia diagnosis and management, as neither country had anemia protocols with clear guidance on integrating preventive MMS and therapeutic iron at the time of study, and both had limited anemia diagnostic capacity. MMS is recommended as a preventive intervention for pregnancy but has insufficient iron (only 30 mg) to meet the treatment requirements for anemia in pregnancy therefore therapeutic iron supplementation is indicated for anemic women. The HCP training reinforced the need to screen for anemia prior to MMS provision to avoid risks of underdosing anemic women [63]. However, this dependence on screening services and the operationalization of anemia management protocols were perceived to delay MMS provision and compromise adherence. These findings highlight a context-specific implementation constraint related to policy and health system capacity, including diagnostics and overall service readiness, which may have been overlooked when IFA was the standard of care. These results may reflect a broader clinical and programmatic challenge, and underscore the need for clear national policies, guidelines, and HCP capacity-building on the management of anemia in countries adopting MMS to ensure consistent and coordinated patient care [15,64].

In addition, in both Pakistan and Nigeria, high workloads and concerns around the additional time required for MMS counseling were perceived by HCPs and PW to constrain service delivery and hinder MMS provision and counseling. This is in line with IR highlighting provider workload, limited resources, and health system constraints as barriers to quality ANC nutrition services, including counselling and supplement provision [65]. There were also some reports of PW receiving MMS late in pregnancy, a finding that may reflect barriers to MMS provision, including challenges related to MMS supply. However, as supported by program monitoring data, MMS supply was regularly available at ANC, especially in comparison to the typical situation for IFA, for which previous reports have highlighted repetitive stockouts and supply challenges [26,66]. Overall, these provision-related barriers reflect the broader programmatic realities of ANC services, while also identifying points that are specific to MMS such as anemia screening and management [66]. Additional barriers to MMS provision included women’s limited agency and lack of support from key household decision-makers. Such barriers, which have also been described in other LMICs [65,67], are particularly relevant to Pakistan and Nigeria [18,26], where women often have limited autonomy over health-care decisions [52,53], and where access to ANC services and related interventions are shaped by household decision-making dynamics. In these settings, PW often require approval or support from husbands or other family members (such as MILs) to attend ANC visits and initiate and continue supplementation [44,68,69].

A key strength of this study is the inclusion of HCP, PW, and influential family members, allowing researchers to capture different perspectives and examine the system, social, and familial factors that may influence MMS provision, acceptability, and adherence practices. Another strength lies in the study’s mixed-methods design. The combination of interviews and FGD data with quantitative findings enabled a more comprehensive understanding of MMS acceptability and consumption behaviors among PW, extending beyond what survey data alone could capture. An additional strength is the use of multiple modalities and measures of adherence, as is recommended, given there is currently no standard definition or broadly accepted measure for adherence to micronutrient supplements [70]. More specifically, the study used both a subjective measure—self-report—and an objective measure—pill count—the latter providing an estimate that does not rely on PW’s memory and minimizes the potential effects of social desirability on adherence measurement [70,71]. Another strength of this study is that MMS introduction in both Pakistan and Nigeria was built on the existing IFA delivery platforms, with strengthened training, monitoring tools, and supply chain functions. This is particularly important, as strengthening national ANC platforms alongside MMS introduction is essential for sustainable scale-up, enabling countries to effectively deliver MMS and advance maternal nutrition outcomes [72].

However, the study findings should be interpreted considering several important limitations. First, the survey did not use a standardized definition or validated tool to assess acceptability, and hence our quantitative data did not capture all relevant acceptability constructs [44,49]. However, the qualitative component complemented these findings and provided rich insights across various domains of acceptability. Second, the lack of consensus and inconsistencies in the definition of adherence may have limited the comparability of our adherence results with those of other studies. Third, the study was not explicitly designed using a specific implementation science framework, but was rather conceptually guided by the WHO/CDC logic model for vitamin and mineral interventions in public health programs [73], alongside constructs from the Health Belief Model. The mixed method approach was therefore informed by both behavioral determinants of supplement use and broader health-system and structural factors relevant to programme implementation. It is important to note that when examining MMS provision, we were not able to explore health system factors in depth (for example, because we only interviewed HCP and not other health system staff such as those working in management). As a result, the system-level focus was mostly limited to care delivery and interactions between HCP and PW within ANC. Fourth, in this study, MMS adherence was assessed using pill counts among women who were pregnant at the time of the survey and self-reported data among all women (pregnant or postpartum). To reduce potential measurement inconsistency, we conducted a sensitivity analysis assessing self-reported adherence among PW only. Although recall-based estimates were slightly lower than pill-count estimates among PW, we did not test for statistical differences as this was beyond the scope of this paper; therefore, the potential for recall bias cannot be excluded. Fifth, as with all interview-based data collection, social desirability bias may have influenced participants’ responses regarding acceptability and adherence practices. To help minimize this bias, data collectors received training focused on neutral interviewing techniques and avoiding judgmental verbal or non-verbal cues. Sixth, the sampling approach of PW may have introduced selection bias and limited the generalizability of the findings, as participants in both Pakistan and Nigeria were recruited from health care provider or facility lists of PW who had received MMS; consequently, PW who did not access ANC, did not receive MMS, or discontinued care were not represented, potentially inflating reported acceptability and adherence. The study also only included public healthcare facilities and HCP and women who received MMS from these facilities/providers and hence does not capture information about private ANC, which may also limit the generalizability of our findings. Furthermore, in Nigeria, MMS was provided to PW free of charge but in Bauchi state, where the study took place, PW are required to pay a nominal amount for IFA. This is an important policy change and must be taken into consideration when interpreting the findings, especially any comparison of provision, acceptability or adherence between MMS and IFA. In addition, our finding that MMS adherence was higher than previously reported national IFA adherence levels, which may reflect improvements in rollout, should be interpreted with caution given the absence of a concurrent comparison group. Finally, this study was limited to specific geographic areas in Pakistan and Nigeria. Because factors shaping MMS acceptability and adherence—such as ANC access, distance to services, and family or HCP influences—vary across regions, the study findings may not be directly generalizable to all districts or states within either country [49].

## 5. Conclusions

This study showed that MMS is widely acceptable to key stakeholders, including PW, their family members, and HCPs, and that it can be effectively delivered through routine ANC. Adherence levels exceeded typical national and regional antenatal supplementation adherence levels; however, continued efforts are needed to achieve ≥90% adherence levels, to maximize benefits on pregnancy and birth outcomes. In this context, the study identified several facilitators of MMS acceptability and adherence, including perceived benefits, trusting relationships with HCPs, and use of habit-forming strategies. Barriers to acceptability and adherence included forgetting, perceived side effects, as well as context-specific sociocultural and health-system factors such as limited women’s agency, misconceptions, and mistrust in the healthcare system. Building on these enablers and addressing these barriers is critical for designing approaches that can strengthen MMS acceptability and adherence in future programs [46]. The study also identified key enablers and barriers related to MMS provision, underscoring the importance of maintaining trust-based communication and quality counseling with PW, addressing logistical constraints to anemia screening and management as well as strengthening the core services including supply chains, to support sustained access and use of MMS. Given that both Pakistan and Nigeria have committed to scaling up MMS nationwide, this study— which incorporates the perspectives of multiple stakeholders—lays the groundwork for developing optimized, context-specific strategies that address identified challenges and opportunities. In doing so, it provides timely insights to inform national scale-up efforts in these two countries, while also offering transferable lessons for other LMICs considering a transition from IFA to MMS as the standard of care for ANC.

## Figures and Tables

**Table 1:** Socio-demographic and ANC characteristics of the survey sample of women in Pakistan and Nigeria.

	Pakistan (N = 1166)	Nigeria (N = 950)
Socioeconomic Characteristics	%	
Age (years)		
<20	12.9	16.7
20–29	68.7	56.0
30–39	14.6	24.4
>40	0.9	2.9
Don’t know	2.7	0
Education		
No education	44.0	65.6
Primary	13.4	16.2
Secondary	32.5	15.4
Higher	10.1	2.8
Pregnancy status at time of survey		
Pregnant	73.2	68.7
Post-partum	26.8	31.3
Primary decision-maker for PW’s healthcare decisions *		
Self	42.8	4.5
Husband	54.9	92.4
Mother-in-law	49.8	---
Father-in-law	7.3	---
Mother	2.4	---
Sister-in-law	2.1	---
Other female family member	0.2	3.0
Living with extended family	84.3	39.6
ANC Characteristics	Mean (range) or %	
Number of ANC visits during the most recent pregnancy	3.9 (1–10)	3.1 (1–8)
Timing of receipt of first MMS bottle		
During first ANC visit	69.7	92.0
During a follow-up ANC visit	30.3	3.8
Other	N/A	4.2
Cadre of healthcare provider who provided the first bottle of MMS		
Pakistan		
Lady Health Worker	91.9	N/A
Lady Health Visitor	6.9	N/A
Women Medical Officer	0.8	N/A
Doctor	0.4	N/A
Nigeria		
Nurse/Midwife/Community Midwife	N/A	11.5
CHEW/JCHEW	N/A	81.6
Other	N/A	6.9

* Respondents could provide more than one answer. Abbreviations: ANC: Antenatal care; CHEW: Community Health Extension Worker; JCHEW: Junior Community Health Extension Worker; MMS: multiple micronutrient supplementation; PW: pregnant woman.

**Table 2:** Measures of acceptability of MMS among key stakeholders in Pakistan and Nigeria.

	Pakistan (N = 1166)	Nigeria (N = 950)
Indicator		
Acceptability of MMS	% (95% CI)	
Perceived Importance		
PW * who state that it is ‘important’ or ‘very important’ to take MMS everyday	93.1 (89.9–95.4)	98.5 (97.8–99.3)
Husbands of PW who state that it is ‘important’ or ‘very important’ to take MMS everyday	97.5 (95.4–98.7)	93.7 (90.7–96.7)
MIL ^ of PW who state that it is ‘important’ or ‘very important’ to take MMS everyday	82.3 (76.9–86.8)	---
Perceived benefits		
PW who can describe at least one benefit of MMS	91.3 (88.5–93.5)	81.5 (79.0–84.0)
Husbands of PW who can describe at least one benefit of MMS	100.0 (100.0–100.0)	69.7 (64.0–75.4)
MIL ^ of PW who can describe at least one benefit of MMS	75.1 (71.2–78.7)	---
HCPs # who can describe at least three benefits of MMS	67.8 (56.5–77.4)	---
Switch to MMS		
HCPs # who describe the transition to MMS as ‘positive’ or ‘very positive’	91.5 (86.2–94.8)	---

* PW and women up to 3 months post-partum who had received MMS >30 days ago from government health care providers ^ or other influential female family members. # In Pakistan these include: women medical officers (WMO), lady health visitors (LHV), and community-based lady health workers (LHW). Abbreviations: CI: confidence interval; HCP: healthcare provider; MIL: mother-in-law; MMS: multiple micronutrient supplementation; PW: pregnant women.

**Table 3:** Facilitators and barriers to MMS acceptability among PW and other stakeholders in Pakistan and Nigeria, and examples of quotes.

	Examples of Quotes	
Facilitators	Pakistan	Nigeria
Perceived benefits	“*During my previous pregnancies, I used to eat soil due to cravings and the bitter taste in my mouth. However, this time, I am taking MMS regularly, and this bad habit of mine has come to an end.*” (PW, age 31)	“*…it would make us healthier along with the fetus. It increases our appetite and gives us more energy…*” (PW, age 25)
Financial accessibility	“*[a] significant advantage is that MMS is provided free of charge to PW, leading to a positive reception*” (HCP; Facility-based WMO)	“*They feel excited, especially when we tell them it’s free, unlike [IFA], which they have to buy.*” (HCP)
Ease of access	“*Many women face restrictions on visiting health facilities or other places. By delivering the MMS bottle directly to their doorstep, we overcome these barriers and ensure that they receive the necessary supplements despite any limitations on their mobility*” (HCP; Community-based LHW)	-----
Trust in the source recommending MMS	“*I already trust her, and when she [HCP] talked about the benefits of these tablets, I felt even more comfortable taking them.*” (PW, age unknown)	-----
Physical and sensory properties of the MMS pills	-----	“*They [PW] told us that when they were taking IFA, they used to vomit or feel the urge to vomit because of its smell, but with MMS, they don’t have those problems at all.*” (HCP) “*IFA has two pills, but MMS is just a single pill; the PW like it better…*” (HCP)
Barriers		
Perceived negative effects	“*My mother-in-law doubted [thinking] there must be something wrong with these tablets…[she] told me that God forbid, these tablets can lead to miscarriage.*” (PW: age 30).	“*When I take it, I feel normal sometimes. At other times, I feel feverish like when I take medication for malaria*…” (PW, age 17)
Previous positive pregnancy experiences without the use of MMS	“*Common arguments from mothers-in-law citing their own experiences of delivering healthy children without … supplement intake.*” (HCP; Facility-based LHV).	“*I was once pregnant and did not take the MMS drug, yet I still delivered safely.*” (PW, age 18)
Mistrust in the system providing the MMS	“…*prevailing mindset that free government-distributed items might be associated with family planning*…” (HCP; Facility-based LHV).	

Abbreviations: HCP: healthcare provider; IFA: iron and folic acid; LHV: lady health visitor; LHW: lady health worker; MMS: multiple micronutrient supplementation; PW: pregnant women; WMO: women medical officer.

**Table 4:** Adherence to antenatal MMS in Pakistan and Nigeria.

	Pakistan (N = 1166)	Nigeria (N = 950)
	% (95% CI) or Mean (±SE)	
Adherence Indicator		
Consumed any MMS tablets	97.4 (95.5–98.5)	99.9 (99.7–100)
Daily adherence to MMS (% of expected tablets consumed) based on pill count (among women who were pregnant at time of survey) ^1^	73.6 (71.2–76.0)	73.6 (71.5–75.7)
Daily adherence to MMS (% of expected tablets consumed) based on self report among women who were pregnant or post-partum at the time of the survey) ^2^	76.9 (74.7–79.1)	72.5 (70.5–74.5)
Daily adherence to MMS (% of expected tablets consumed) based on self-report (among women who were pregnant at time of survey) ^1^	71.0 (68.3–73.7)	73.2 (71.1–75.3)
Consumed ≥75% of expected MMS tablets, based on pill count (among women who were pregnant at time of survey) ^1^	60.4 (56.7–64.0)	55.1 (51.2–59.0)
Consumed ≥75% of expected MMS tablets, based on self report (among women who were pregnant or post-partum at the time of the survey) ^2^	66.3 (63.0–69.6)	55.4 (52.0–58.7)
Consumed ≥75% of expected MMS tablets, based on self report (among women who were pregnant at time of survey) ^1^	56.8 (52.7–60.9)	53.1 (49.1–57.3)
Consumed ≥90% of expected MMS tablets, based on pill count (among women who were pregnant at time of survey) ^1^	48.6 (44.8–52.3)	40.1 (36.2–44.0)
Consumed ≥90% of expected MMS tablets, based on self report (among women who were pregnant or post-partum at the time of the survey ^2^	57.3 (53.8–60.8)	41.4 (38.1–44.8)
Consumed ≥90% of expected MMS tablets, based on self report (among women who were pregnant at time of survey) ^1^	45.6 (41.5–50.0)	36.4 (32.4–40.3)
Patterns of adherence		
Stopped taking MMS at some point during pregnancy	28.3 (25.7–31.0)	35.2 (32.0–38.0)
Stopped and restarted taking MMS (among all PW)	20.8 (18.4–23.1)	26.8 (24.0–29.7)
Stopped and did not restart taking MMS (among all PW)	7.6 (6.0–9.1)	8.3 (6.6–10.1)
Days stopped before restarting (among women who reported stopping and restarting only)	7.48 (±1.0)	5.7 (±0.9)
Reason why PW stopped taking MMS *		
Forgot to take	62.1 (57.0–67.0)	72.5 (68.0–77.0)
Perceived side effects	36.6 (31.0–42.0)	11.1 (7.7–14.5)
Ran out of MMS before the next ANC visit	1.2 (0.0–2.0)	0.6 (0.0–1.0)
Female family member/mother-in-law advised not to take	0.6 (0.0–1.0)	0.9 (0.0–2.0)
Diagnosed with anemia	3.4 (1.0–5.0)	0.3 (0.0–1.0)
Stopped because pregnancy ended	N/A	4.8 (2.0–7.0)
Husband advised not to take	0.9 (0.0–2.0)	0.3 (0.0–1.0)
HCP advised not to take	0.6 (0.0–1.0)	N/A
Other	4.0 (2.0–6.0)	1.2 (0.0–2.0)

^1^ Among the sample of women who were pregnant at the time of survey: n = 854 in Pakistan; n = 653 in Nigeria; ^2^ Among the sample of women who were pregnant or post-partum at the time of survey. n = 1166 in Pakistan; n = 950 in Nigeria; * Respondents could provide more than one answer.; Abbreviations: ANC: antenatal care; CI: confidence interval; HCP: healthcare provider; MMS: multiple micronutrient supplementation; PW: pregnant women; SE: standard error.

**Table 5:** Facilitators and barriers to MMS adherence among PW and other stakeholders in Pakistan and Nigeria, and examples of quotes.

	Examples of Quotes	
Facilitators	Pakistan	Nigeria
Perceived benefits	“*In my previous pregnancy, I experienced anemia … This time, with MMS, my HB is stable.*” (PW, age 33)	“*The supplement improves our health and that of our baby, so one has to remember to take it.*” (PW, age 30)
Using daily reminder techniques	“*The MMS bottle is strategically placed on the table. Every day, as I pass by, I’m prompted to take it. Even when collecting things from the table, the sight of the MMS bottles serves as a reminder to ensure I don’t miss my daily dose*…” (PW, age unknown)	“*I usually keep it near my pap [sic] every morning. When it’s time to eat, I just take my food and the supplement.*” (PW, age 17)
Encouragement by family members	“*…I started taking them regularly as my sister also advised me to take them.*” (PW, age 20)	“*If your husband pays attention and realizes that you tend to forget your medication, he will always remind you to take it.*” (PW, age unknown)
Knowledge related to MMS	“*The information provided by the LHW was immensely beneficial. Her explanation about the importance and advantages of MMS has encouraged me to take the tablets regularly.*” (PW, age unknown)	---
Strategies to mitigate side effects	“*I took the medicines at night and faced stomach issues. If she had not told me to take them during the meal, I would have skipped taking these tablets.*” (PW, age 25)	---
Fear or shame-based beliefs about lack of adherence	---	“*…they [HCP] also know if the mother is not taking MMS regularly, when she gives birth the baby will not come out as clean as they expect.*” (PW, age 22)
Barriers		
Perceived negative effects	“*I wasn’t comfortable with these MMS tablets as they were causing nausea. So, I took a break from these tablets every two days, giving myself a one-day break.*” (PW, age 20)	“*Some women mistakenly believe that taking it [MMS] will make their fetus bigger, leading to a more difficult childbirth. So, they refuse to take it.*” (PW, age unknown)
Disruptions to daily routines	“*There were instances where I forgot to take my daily dose of MMS, particularly when I was away visiting my parents or attending events like weddings or funerals.*” (PW, age unknown)	---

Abbreviations: HCP: healthcare provider; LHW: lady health worker; MMS: multiple micronutrient supplementation; PW: pregnant women.

**Table 6:** Facilitators and barriers to MMS provision through public ANC in Pakistan and Nigeria, and examples of quotes.

	Examples of Quotes	
Facilitators	Pakistan	Nigeria
Open communication and a trusting relationship between HCP and PW	“*Our Lady Health Worker is good for us. We prefer her to supply these MMS tablets to us. She dedicates ample time to us, explaining the usage and benefits of these tablets.*” (PW, age unknown)	“*They [HCPs] provide free maternal supplements and ANC services. They always want the best for us, which is something that wasn’t available in the past. They take care of us properly and respect us very much.*” (PW, age 22)
Accessibility of MMS	“*We appreciate the convenience of having them [MMS] brought to our homes, as we may not have sufficient time to visit the health facility and endure long queues to receive these MMS tablets*” (PW, age unknown) “*Women face restrictions on visiting health facilities or other places (mobility issues). We overcome the mobility issue by delivering the MMS bottle directly to their doorstep.*” (HCP; Community-based LHW)	“*When my supply runs out, I return to get more. There has never been a time I went and it wasn’t available.*” (PW, age unknown)
Trained HCPs	----	“*The training I received … was very beneficial. I learned how to properly educate PW about MMS, how to explain its benefits, and how to monitor their usage.*” (HCP)
Barriers		
Heavy workload among HCPs	“*In hospitals, there is often a high volume of patients, and the staff may not provide sufficient time for information about MMS tablets.*” (PW, age unknown)	“*Sometimes there are too many people at the health facility, so the healthcare worker may not have enough time to attend to everyone at once.*” (PW, age unknown)
Anemia diagnosis and management	“*At Basic Health Unit, there is no laboratory for essential tests available here. Given my recurrent issue of anemia during pregnancy, this lack of testing facilities is a significant concern for me.*” (PW, age unknown). “*First bottle [is provided in] 3rd or 4th months by assessing the physical appearance and health. HB [hemoglobin] levels are considered.* (HCP)	“*IFA can be taken by any pregnant woman, whereas MMS requires a certain blood level threshold before it can be used.*” (HCP)
Limited agency among PW	“*Husbands or other family members may not permit them to attend follow-up visits, thereby impeding the continuity of care and provision of the second bottle of MMS.*” (HCP; Facility-based LHV)	“*[PW] do face challenges. Not having transport fare is one of them, and lack of support from their families is another significant challenge.*” (HCP)
Delayed initiation of ANC	“*For late comers, HCPs provide first bottle in the 7th or 8th months.*” (HCP).	“*Some women wait until seven months before coming for ANC.*” (PW, age unknown)
Sociocultural norms	“*PW hide pregnancy due to conservative families especially mother in-laws.*” (HCP)	“*…some families strictly rely on traditional medicine and do not believe in conventional hospital treatment.*” (Husband, age 40)
Inconsistent MMS supply	“*…the supply of these tablets was delayed…Maybe the supply of these tablets arrived late to our Lady Health Worker.*” (PW, age 32) “*It was in my ninth month when she finally provided me with the first MMS bottle.*” (PW, age 32)	“*Sometimes stock runs out—for instance, since February, it has been unavailable.*” (HCP)
Limited coordination and communication among/between community- and facility- based platforms	“*[LHW] doesn’t stay in touch with us … consequently, it seems there is a disconnect as our work fields are not entirely relevant to each other… there is minimal or no coordination between LHWs and LHVs.*” (HCP; Facility-based LHV)	---

Abbreviations: ANC: antenatal care; HCP: healthcare provider; IFA: iron and folic acid; LHV: lady health visitor; LHW: lady health worker; MMS: multiple micronutrient supplementation; PW: pregnant women.

## Data Availability

The data presented in this study are available on reasonable request from the corresponding author due to privacy and ethical reasons.

## Abbreviations

The following abbreviations are used in this manuscript:

ANC	Antenatal care
CAPI	Computer-assisted personal interviewing
CHEW	Community Health Extension Worker
CI	Confidence interval
ERC	Ethical review committee
FGDs	Focus group discussions
GoN	Government of Nigeria
GoP	Government of Pakistan
HCP	Healthcare provider
IDI	In-depth interviews
IFA	Iron and folic acid
IFFM	Influential female family member
IR	Implementation research
JCHEW	Junior Community Health Extension Worker
LBW	Low birth weight
LGAs	Local government area
LHV	Lady health visitors
LHW	Lady health worker
LMICs	Low- and middle-income countries
MIL	Mother-in-law
MMS	Multiple micronutrient supplementation
NDHS	Nigeria Demographic and Health Surveys
NFCS-MN	Nigeria National Food Consumption and Micronutrient Survey
PW	Pregnant women
SE	Standard error
SGA	Small-for-gestational-age
UC	Union councils
UNIMMAP	United Nations International Multiple Micronutrient Antenatal Preparation
WHO	World Health Organization
WMO	Women medical officer
WRA	Women of reproductive age

## References

[1] Victora C.G., Christian P., Vidaletti L.P., Gatica-Domínguez G., Menon P., Black R.E. (2021). Revisiting maternal and child undernutrition in low-income and middle-income countries: Variable progress towards an unfinished agenda. Lancet.

[2] Bourassa M.W., Osendarp S.J., Adu-Afarwuah S., Ahmed S., Ajello C., Bergeron G., Black R., Christian P., Cousens S., de Pee S. (2019). Review of the evidence regarding the use of antenatal multiple micronutrient supplementation in low-and middle-income countries. Ann. N. Y. Acad. Sci..

[3] Gernand A.D., Schulze K.J., Stewart C.P., West K.P., Christian P. (2016). Micronutrient deficiencies in pregnancy worldwide: Health effects and prevention. Nat. Rev. Endocrinol..

[4] World Health Organization, Study Group on Iron Deficiency Anaemia, World Health Organization (1959). Iron Deficiency Anaemia: Report of a Study Group.

[5] World Health Organization (2012). Guideline: Daily Iron and Folic Acid Supplementation in Pregnant Women.

[6] Ajello C.A., Atwater J., de Lange J. (2024). Expert Consensus on an Open-Access UNIMMAP MMS Product Specification: 2024 revision. Ann. N. Y. Acad. Sci..

[7] Smith E.R., Shankar A.H., Wu L.S., Aboud S., Adu-Afarwuah S., Ali H., Agustina R., Arifeen S., Ashorn P., Bhutta Z.A. (2017). Modifiers of the effect of maternal multiple micronutrient supplementation on stillbirth, birth outcomes, and infant mortality: A meta-analysis of individual patient data from 17 randomised trials in low-income and middle-income countries. Lancet Glob. Health.

[8] Keats E.C., Haider B.A., Tam E., Bhutta Z.A. (2019). Multiple-micronutrient supplementation for women during pregnancy. Cochrane Database Syst. Rev..

[9] Engle-Stone R., Kumordzie S.M., Meinzen-Dick L., Vosti S.A. (2019). Replacing iron-folic acid with multiple micronutrient supplements among pregnant women in Bangladesh and Burkina Faso: Costs, impacts, and cost-effectiveness. Ann. N. Y. Acad. Sci..

[10] Kashi B., Godin C.M., Kurzawa Z.A., Verney A.M., Busch-Hallen J.F., De-Regil L.M. (2019). Multiple micronutrient supplements are more cost-effective than iron and folic acid: Modeling results from 3 high-burden Asian countries. J. Nutr..

[11] Gernand A.D. (2019). The upper level: Examining the risk of excess micronutrient intake in pregnancy from antenatal supplements. Ann. N. Y. Acad. Sci..

[12] Black R.E., Dewey K.G. (2019). Benefits of supplementation with multiple micronutrients in pregnancy. Ann. N. Y. Acad. Sci..

[13] Wang D., Liu E., Perumal N., Partap U., Cliffer I.R., Costa J.C., Wang M., Fawzi W.W., Adu-Afarwuah S., Ashorn P. (2025). The effects of prenatal multiple micronutrient supplementation and small-quantity lipid-based nutrient supplementation on small vulnerable newborn types in low-income and middle-income countries: A meta-analysis of individual participant data. Lancet Glob. Health.

[14] World Health Organization (2020). WHO Antenatal Care Recommendations for a Positive Pregnancy Experience. Nutritional Interventions Update: Multiple Micronutrient Supplements During Pregnancy.

[15] Gomes F., Agustina R., Black R.E., Christian P., Dewey K.G., Kraemer K., Shankar A.H., Smith E.R., Thorne-Lyman A., Tumilowicz A. (2022). Multiple micronutrient supplements versus iron-folic acid supplements and maternal anemia outcomes: An iron dose analysis. Ann. N. Y. Acad. Sci..

[16] Peters D.H., Tran N.T., Adam T. (2013). Implementation Research in Health: A Practical Guide.

[17] Tropical Disease Research Massive Open Online Course (MOOC) on Implementation Research: Infectious Diseases of Poverty. https://tdr.who.int/home/our-work/strengthening-research-capacity/massive-open-online-course-(mooc)-on-implementation-research.

[18] Siekmans K., Roche M., Kung’u J.K., Desrochers R.E., De-Regil L.M. (2018). Barriers and enablers for iron folic acid (IFA) supplementation in pregnant women. Matern. Child. Nutr..

[19] Black R.E., Victora C.G., Walker S.P., Bhutta Z.A., Christian P., De Onis M., Ezzati M., Grantham-McGregor S., Katz J., Martorell R. (2013). Maternal and child undernutrition and overweight in low-income and middle-income countries. Lancet.

[20] Heidkamp R.A., Piwoz E., Gillespie S., Keats E.C., D’Alimonte M.R., Menon P., Das J.K., Flory A., Clift J.W., Ruel M.T. (2021). Mobilising evidence, data, and resources to achieve global maternal and child undernutrition targets and the Sustainable Development Goals: An agenda for action. Lancet.

[21] National Institute of Population Studies (NIPS) [Pakistan], ICF (2019). Pakistan Demographic and Health Survey 2017-18.

[22] Ministry of National Health Services Regulations and Coordination, United Nations Children’s Fund (UNICEF), Aga Khan University (2018). Pakistan National Nutrition Survey 2018.

[23] Fayehun O., Asa S. (2020). Abnormal birth weight in urban Nigeria: An examination of related factors. PLoS ONE.

[24] National Population Commission (NPC), ICF (2019). Nigeria Demographic and Health Survey 2018.

[25] Federal Government of Nigeria (FGoN), The International Institute of Tropical Agriculture (IITA) (2024). Nigeria National Food Consumption and Micronutrient Survey (NFCS-MN) 2021.

[26] Ministry of National Health Services Regulations and Coordination, United Nations Children’s Fund (UNICEF), Nutrition International (2022). Iron Folic Acid (IFA) Bottleneck Analysis Report.

[27] United Nations Global Marketplace Folic Acid Supply Chain Strengthening. https://www.ungm.org/Public/Notice/80365?utm_source.

[28] Smith E.R., Gomes F., Adu-Afarwuah S., Aguayo V.M., El Arifeen S., Bhutta Z.A., Caniglia E.C., Christian P., Devakumar D., Dewey K.G. (2025). Contribution of maternal adherence to the effect of multiple micronutrient supplementation during pregnancy: A systematic review and individual participant data meta-analysis. Adv. Nutr..

[29] Ministry of National Health Services Regulations and Coordination, United Nations Children’s Fund (UNICEF) (2022). Pakistan Maternal Nutrition Strategy 2022—2027.

[30] Nutrition International (2023). Multiple Micronutrient Supplementation Implementation Research in Nigeria: Optimizing Adherence to Maternal Multiple Micronutrient Supplementation. https://nutritionintl.org/learning-resource/mms-implementation-research-nigeria/.

[31] Federal Ministry of Health (2021). National Guidelines for the Prevention and Control of Micronutrient Deficiencies in Nigeria.

[32] Meh C., Thind A., Ryan B., Terry A. (2019). Levels and determinants of maternal mortality in northern and southern Nigeria. BMC Pregnancy Childbirth.

[33] Higazi A., Lar J. (2015). Articulations of belonging: The Politics of ethnic and religious pluralism in Bauchi and Gombe states, north-east Nigeria. Africa.

[34] Qureshi R.N., Sheikh S., Khowaja A.R., Hoodbhoy Z., Zaidi S., Sawchuck D., Vidler M., Bhutta Z.A., Von Dadeslzen P., Group C.W. (2016). Health care seeking behaviours in pregnancy in rural Sindh, Pakistan: A qualitative study. Reprod. Health.

[35] Idris I.B., Hamis A.A., Bukhori A.B.M., Hoong D.C.C., Yusop H., Shaharuddin M.A.-A., Fauzi N.A.F.A., Kandayah T. (2023). Women’s autonomy in healthcare decision making: A systematic review. BMC Womens Health.

[36] Pakistan Bureau of Statistics Khyber Pakhtunkhwa Insight: 7th Population and Housing Census, First Digital Census of Pakistan. https://www.pbs.gov.pk/sites/default/files/population/2023/material/kp_insight.pdf.

[37] Guest G., Fleming P., Guest G., Namey E. (2015). Mixed methods research. Public Health Research Methods.

[38] Merriam S., Tisdell E. (2015). Qualitative Research: A Guide to Design and Implementation.

[39] Lambert S.D., Loiselle C.G. (2008). Combining individual interviews and focus groups to enhance data richness. J. Adv. Nurs..

[40] Braun V., Clarke V. (2022). Conceptual and design thinking for thematic analysis. Qual. Psychol..

[41] Ahmed S.K., Mohammed R.A., Nashwan A.J., Ibrahim R.H., Abdalla A.Q., Ameen B.M.M., Khdhir R.M. (2025). Using thematic analysis in qualitative research. J. Med. Surg. Public Health.

[42] Ummair M., Durrani A., Jamshaid M., Amjad M., Gul M., Khan J.Z., Khan A.Q. (2025). Compliance With Iron and Folic Acid Supplements Among Pregnant Women Attending Tertiary Care Hospital in the District of Peshawar. Cureus.

[43] Fite M.B., Roba K.T., Oljira L., Tura A.K., Yadeta T.A. (2021). Compliance with Iron and Folic Acid Supplementation (IFAS) and associated factors among pregnant women in Sub-Saharan Africa: A systematic review and meta-analysis. PLoS ONE.

[44] Kissell M.C., Pereira C., Gomes F., Woldesenbet K., Tessema M., Kelemu H., Noor R., Escubil L., Panicker A., Mishra A. (2025). Acceptability of Antenatal Multiple Micronutrient Supplementation (MMS) Compared to Iron and Folic Acid (IFA) Supplementation in Pregnant Individuals: A Narrative Review. Nutrients.

[45] Abebe F., Kidanemariam Y.T., Tsegaw M., Birhanu Z., Abdi A., Chitekwe S., Sharma R., Getachew H., Noor R., Aden A.H. (2025). Acceptance of multiple micronutrient supplementations (MMS) and iron and folic acid supplement utilisation among pregnant and lactating women in the rural part of Ethiopia, 2022: A cross-sectional study. BMJ Open.

[46] Clermont A., Kodish S.R., Matar Seck A., Salifou A., Rosen J., Grais R.F., Isanaka S. (2018). Acceptability and utilization of three nutritional supplements during pregnancy: Findings from a longitudinal, mixed-methods study in Niger. Nutrients.

[47] Aguayo V.M., Koné D., Bamba S.I., Diallo B., Sidibé Y., Traoré D., Signé P., Baker S.K. (2005). Acceptability of multiple micronutrient supplements by pregnant and lactating women in Mali. Public Health Nutr..

[48] Ba A., Fox M.J., Keita A.M., Hurley K.M., King S.E., Sow S., Diarra K., Djiteye M., Kanté B.S., Coulibaly M. (2025). Qualitative evaluation of a package of implementation strategies codesigned to support the introduction of multiple micronutrient supplementation (MMS) for pregnant women in Bamako, Mali. Matern. Child Nutr..

[49] Sauer C., Hoang M.-A., Kroeun H., Gupta A.S., Ngik R., Sokchea M., Labonté J.M., Chea M., Klemm R., Mishra A. (2025). Assessing the adherence and acceptability to iron and folic acid compared with multiple micronutrient supplements during pregnancy: A cluster-randomized noninferiority trial in Cambodia. Am. J. Clin. Nutr..

[50] Horino M., Habash R., Seita A., Al-Khatib L., Al Rahahleh T., Kraemer K., Hurley K., West K.P. (2024). Systems Trial of Antenatal Multiple Micronutrient Supplements (MMS) Versus Iron-Folic Acid in a UN Health Care System Serving Palestinian Camps in Jordan. Curr. Dev. Nutr..

[51] Abidah N., Sumarmi S. (2024). A Comparison of Adherence Levels of Pregnant Women to Consuming Multiple Micronutrient Supplements and Iron Folic Acid at Mulyorejo Public Health Center, Surabaya. Amerta Nutr..

[52] Khan B.A.A., Mahmood H., Ahmed J.M., Anwar B., Muhammad A., Jabeen R. (2025). Exploring challenges in accessing primary healthcare for pregnant women in Pakistan: A qualitative descriptive study. BMC Health Serv. Res..

[53] Rizvi Jafree S., Barlow J. (2023). Systematic review and narrative synthesis of the key barriers and facilitators to the delivery and uptake of primary healthcare services to women in Pakistan. BMJ Open.

[54] Martin S.L., Omotayo M.O., Chapleau G.M., Stoltzfus R.J., Birhanu Z., Ortolano S.E., Pelto G.H., Dickin K.L. (2017). Adherence partners are an acceptable behaviour change strategy to support calcium and iron-folic acid supplementation among pregnant women in Ethiopia and Kenya. Matern. Child. Nutr..

[55] Stawarz K., Cox A., Blandford A. Understanding the role of contextual cues in supporting the formation of medication-taking habits. Proceedings of the 2nd Behaviour Change Conference: Digital Health and Wellbeing.

[56] Sauer C., Sokchea M., Sreang S., Kroeun H., Hun V., Sen Gupta A., Rattana K., Chea M., Hoang M.-A. (2026). Exploring Behavioral Interventions to Enhance Adherence to Multiple Micronutrient Supplementation Among Pregnant Women in Cambodia: A Mixed-Methods Study. Nutrients.

[57] Lassi Z.S., Bhutta Z.A. (2015). Community-based intervention packages for reducing maternal and neonatal morbidity and mortality and improving neonatal outcomes. Cochrane Database Syst. Rev..

[58] World Health Organization (2018). WHO Recommendations on Health Policy and System Support to Optimize Community Health Worker Programme.

[59] Prost A., Colbourn T., Seward N., Azad K., Coomarasamy A., Copas A., Houweling T.A., Fottrell E., Kuddus A., Lewycka S. (2013). Women’s groups practising participatory learning and action to improve maternal and newborn health in low-resource settings: A systematic review and meta-analysis. Lancet.

[60] Anato A., Reshid M. (2025). Effect of nutrition education and iron-folic acid supplementation on anemia among pregnant women in Ethiopia: A quasi-experimental study. Sci. Rep..

[61] Marsh A.D., Muzigaba M., Diaz T., Requejo J., Jackson D., Chou D., Cresswell J.A., Guthold R., Moran A.C., Strong K.L. (2020). Effective coverage measurement in maternal, newborn, child, and adolescent health and nutrition: Progress, future prospects, and implications for quality health systems. Lancet Glob. Health.

[62] Berhanu A.T., Defar A., Taye G., Daba A.K., Alemayehu S., Tessema B., Zenebe K., Opondo C., Tollera G., Hailu M. (2025). Piloting Multiple Micronutrient Supplementation Within the Routine Antenatal Care System in Ethiopia: Insights From Stakeholders. Matern. Child Nutr..

[63] UNICEF (2021). Interim Country-Level Decision-Making Guidance for Introducing Multiple Micronutrient Supplementation for Pregnant Women.

[64] Smith E.R., Muhihi A., Wylie B.J., Mugusi S., Aboud S., Bakari M., Fawzi W., Kinyogoli S., Oakley E.M., Pan Q. (2025). Multiple micronutrient supplementation for maternal anemia prevention (MMS-MAP): An individually randomized trial of higher-dose iron (60 mg, 45 mg) compared to low-dose iron (30 mg) in multiple micronutrient supplements in pregnancy. Trials.

[65] Labonté J.M., Hoang M.A., Panicker A., Kroeun H., Sokchea M., Sambo S., Sokhal V., Sauer C., Chea M., Karakochuk C.D. (2025). Exploring factors affecting adherence to multiple micronutrient supplementation during pregnancy in Cambodia: A qualitative analysis. Matern. Child Nutr..

[66] Siekmans K., Tinkler J., Raguenaud M. (2019). Implementing antenatal iron and folic acid supplementation programs: Barriers and enablers in 7 countries. Glob. Health Sci. Pract..

[67] Silubonde T.M., Draper C.E., Baumgartner J., Ware L.J., Smuts C.M., Norris S.A. (2022). Barriers and facilitators of micronutrient supplementation among non-pregnant women of reproductive age in Johannesburg, South Africa. PLoS Glob. Public Health.

[68] Nisar Y.B., Alam A., Aurangzeb B., Dibley M.J. (2014). Perceptions of antenatal iron-folic acid supplements in urban and rural Pakistan: A qualitative study. BMC Pregnancy Childbirth.

[69] Azuh D., Fayomi O., Ajayi L. (2015). Socio-cultural factors of gender roles in women’s healthcare utilization in Southwest Nigeria. Open J. Soc. Sci..

[70] Burleson J., Stephens D.E., Rimal R.N. (2025). Adherence definitions, measurement modalities, and psychometric properties in hiv, diabetes, and nutritional supplementation studies: A scoping review. Patient Prefer. Adherence.

[71] Stirratt M.J., Dunbar-Jacob J., Crane H.M., Simoni J.M., Czajkowski S., Hilliard M.E., Aikens J.E., Hunter C.M., Velligan D.I., Huntley K. (2015). Self-report measures of medication adherence behavior: Recommendations on optimal use. Transl. Behav. Med..

[72] BMGF, CIFF, ECF, Kirk Humanitarian (2024). Healthier Pregnancies and Brighter Futures for Mothers and Babies. A Global Investment Roadmap for Multiple Micronutrient Supplementation. https://kirkhumanitarian.org/wp-content/uploads/2024/07/MMS-Investment-Roadmap-Digital-B-1.pdf.

[73] De-Regil L.M., Pena-Rosas J.P., Flores-Ayala R., del Socorro Jefferds M.E. (2014). Development and use of the generic WHO/CDC logic model for vitamin and mineral interventions in public health programmes. Public Health Nutr..

